# Healthcare Professional in the Loop (HPIL): Classification of Standard and Oral Cancer-Causing Anomalous Regions of Oral Cavity Using Textural Analysis Technique in Autofluorescence Imaging

**DOI:** 10.3390/s20205780

**Published:** 2020-10-12

**Authors:** Muhammad Awais, Hemant Ghayvat, Anitha Krishnan Pandarathodiyil, Wan Maria Nabillah Ghani, Anand Ramanathan, Sharnil Pandya, Nicolas Walter, Mohamad Naufal Saad, Rosnah Binti Zain, Ibrahima Faye

**Affiliations:** 1 Center for Intelligent Medical Electronics, Department of Electronic Engineering, School of Information Science and Technology, Fudan University, Shanghai 200433, China; 17110720061@fudan.edu.cn; 2 Innovation Division Technical University of Denmark, 2800 Lyngby, Denmark; ghayvat@gmail.com; 3 Oral Diagnostic Sciences, Faculty of Dentistry, SEGi University, Jalan Teknologi, Kota Damansara, Petaling Jaya 47810, Selangor, Malaysia; anithakrishnan@segi.edu.my; 4 Oral Cancer Research and Coordinating Centre, Faculty of Dentistry, University of Malaya, Kuala Lumpur 50603, Malaysia; nabilah_wm@um.edu.my (W.M.N.G.); drranand@um.edu.my (A.R.); profrosnah@mahsa.edu.my (R.B.Z.); 5 Department of Oral and Maxillofacial Clinical Sciences, Faculty of Dentistry, University of Malaya, Kuala Lumpur 50603, Malaysia; 6 Symbiosis Centre for Applied Artificial Intelligence and CSE Dept, Symbiosis International (Deemed) University, Pune 412115, Maharashtra, India; sharnil.pandya@scaai.siu.edu.in; 7 Department of Electrical and Electronic Engineering, Universiti Teknologi PETRONAS, Bandar Seri Iskandar 32610, Perak, Malaysia; walter.nicolas.pro@gmail.com (N.W.); naufal_saad@utp.edu.my (M.N.S.); 8 MAHSA University, Dean Office, Level 9, Dental Block, Bandar Saujana Putra, Jenjarom 42610, Selangor, Malaysia; 9 Department of Fundamental and Applied Sciences, Universiti Teknologi PETRONAS, Bandar Seri Iskandar 32610, Perak, Malaysia

**Keywords:** oral mucosal cancer, oral potentially malignant disorders (OPMD), oral cavity mucosal lesions, autofluorescence imaging, texture analysis, VELscope^®^

## Abstract

Oral mucosal lesions (OML) and oral potentially malignant disorders (OPMDs) have been identified as having the potential to transform into oral squamous cell carcinoma (OSCC). This research focuses on the human-in-the-loop-system named Healthcare Professionals in the Loop (HPIL) to support diagnosis through an advanced machine learning procedure. HPIL is a novel system approach based on the textural pattern of OML and OPMDs (anomalous regions) to differentiate them from standard regions of the oral cavity by using autofluorescence imaging. An innovative method based on pre-processing, e.g., the Deriche–Canny edge detector and circular Hough transform (CHT); a post-processing textural analysis approach using the gray-level co-occurrence matrix (GLCM); and a feature selection algorithm (linear discriminant analysis (LDA)), followed by k-nearest neighbor (KNN) to classify OPMDs and the standard region, is proposed in this paper. The accuracy, sensitivity, and specificity in differentiating between standard and anomalous regions of the oral cavity are 83%, 85%, and 84%, respectively. The performance evaluation was plotted through the receiver operating characteristics of periodontist diagnosis with the HPIL system and without the system. This method of classifying OML and OPMD areas may help the dental specialist to identify anomalous regions for performing their biopsies more efficiently to predict the histological diagnosis of epithelial dysplasia.

## 1. Introduction

**Figure 1:**
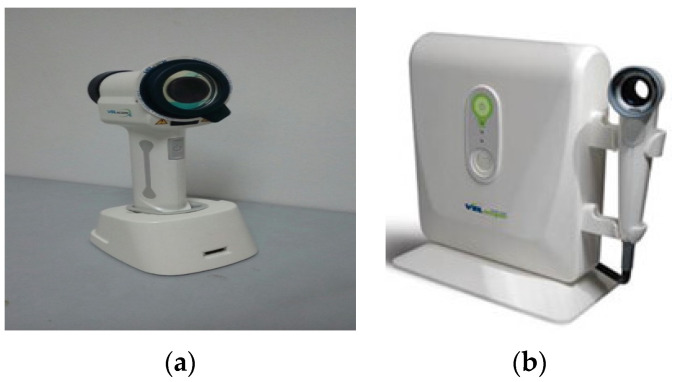
Autofluorescence devices: (**a**) 3rd generation VELscope^®^; (**b**) 2nd generation VELscope^®^.

Oral potentially malignant disorders (OPMDs) are one of the severe health issues across the globe [1]. OPMDs include oral leukoplakia [2], oral erythroplakia [3,4], oral submucous fibrosis, oral lichen planus [5], and oral lichenoid reaction [6], Other oral mucosal lesions (OML) include non-specific ulcerations, erythematous lesions, abscesses, median rhomboid glossitis, frictional keratosis, and pyogenic granuloma. The detection and diagnosis of these OPMDs as early as possible are essential for the dental specialist, as these anomalies might transform into oral cancer [7,8,9,10]. At the advanced stage, it is more challenging to cure oral cancer. The accepted method for the detection of OML and OPMDs is the conventional oral examination (COE) (visual inspection) [11,12]. The COE is exceedingly sensitive in detecting vicissitudes in the oral cavity because of the easy visibility of various oral cavity structures. However, the predictions of which COE-identified lesions will progress to oral cancer is dependent on the histopathological findings of epithelial dysplasia, as reported by an oral pathologist in biopsy tissue [13]. Currently, along with the use of the COE, clinicians/dental specialists use different additional examinations, e.g., the toluidine blue test (TB) [14], ViziLite^®^ plus [15], VELscope^®^ as shown in Figure 1, and Identafi^®^ [16] as an adjunct to the COE in locating the area/areas to increase the predictive value for epithelial dysplasia. The histopathological diagnosis of epithelial dysplasias is currently the gold standard, as they have a higher potential for malignant change as compared to COE-identified OPMDs [17].

With the aid of these added tools, the precision in detecting areas that predict epithelial dysplasia in these oral lesions may improve. Although these adjunct tools may help the clinicians/dental specialists in detecting potential areas with epithelial dysplasia, the examination of the images relies on clinicians′ experience to discriminate amongst the potentially standard and anomalous regions [17,18]. The autofluorescent imaging device (Identafi, VELscope) permits the dentist to record video frames that are used for more detailed examinations.

Texture analysis is one of the crucial aspects of the vision system for differentiating between surfaces and objects. A surface is usually defined as rough (having a massive difference between high and low points) or silky (having little difference between low and high points), and a bumpy surface refers to touch [19]. For three decades, in digital image processing, texture has referred to visual configurations or the spatial organization of pel that regional intensity or color cannot explain adequately. Texture provides a better explanation of the content of the image structure as compared to an intensity illustrator, e.g., the average gray level, minimum/maximum gray level, or histogram of the local region. The texture appears to be a significant feature for an automated and semi-automated explanation of digital images for detecting the region of interest (ROI). It has a history of more than three decades in the field of educational, biomedical [20,21], military [22], commercial [23], web, and satellite image classification [24].

Medical image processing helps the clinicians by analyzing, in an objective way, the various kinds of images and different textures associated. As a result, the disease areas are identified more accurately from the standard ones. In this paper, the statistical behavior of the texture that occurs in the autofluorescence images is discussed to identify anomalous areas inside the oral cavity based on autofluorescence imaging [25]. In the case of biomedical images, textures occur randomly in the images but possess specific characteristics due to the symmetry in each body part of a living organism. This study proposes the use of the gray-level co-occurrence matrix (GLCM) parameters as features to identify the anomalous areas. It has to be mentioned that, to our knowledge, no image processing techniques based on textural pattern analysis have been attempted to date on the autofluorescence images from clinicians’ examinations using a machine learning approach. The main contribution of this article is as follows:

1. The proposed Healthcare Professional in the loop (HPIL) model acts as an aided tool for periodontists by automatically analyzing the VELscope^®^ image of an oral cavity to find the ROI more precisely.
2. A texture-based machine learning algorithm using VELscope^®^ images to discriminate OPMD and OML regions from a normal oral cavity.
3. The design of a Graphical User Interface (GUI) to assist clinicians in the classification of OPMDs.

The remainder of the paper is organized as follows: Section 2 describes the background of the algorithms used in this paper; Section 3 details the approach proposed, dataset information, and statistical testing criteria for result verification. Section 4 and Section 5 present the results and discussion, respectively, and Section 6 concludes the paper.

## 2. Background

### 2.1. State-of-the-Art Techniques and Necessity of Research

Screening for oral cavities implies searching for OPMDs and OML, typically before symptoms occur. Traditionally, the following steps have been followed by clinicians for screening and diagnosis inside the oral cavity. **1. Determining the background history of existing disease (if any):** (a) the beginning, place, strength, occurrence, and period; (b) any irritation or discharges; (c) whether the disease has improved, remained constant, or worsened over the period. **2. Medical and drugs history (if any):** (a) medical circumstances; (b) medications and antipathies; (c) tobacco and alcohol history (nature and time). **3. Medical examination:** (a) extra mouth-cavity screening; (b) an intraoral check-up; (c) lesion screening using adjunctive visual tools such as direct fluorescence. **4. Differential analysis. 5. Diagnostic tests:** (a) a biopsy (if needed)**. 6. Definitive diagnosis. 7. Suggested management** [26]. With the advent of various diagnostic modalities, today, clinicians are using numerous approaches for the early diagnosis of OPMDs and OML [27]. **ViziLite:** ViziLite is an adjunctive tool working on the principle of tissue reflectance that has been used for the screening of the oral mucosa for “‘acetowhite” premalignant and malignant lesions. In recent times, a tissue reflectance-based screening device has been used for the oral cavity examination and is currently available as ViziLite [28]. **Identafi^®^:** The deep-penetrating multispectral lighting (three wavelengths) of the Identafi^®^ enhances the early detection of abnormalities inside the oral cavity that might be cancerous. It uses the fluorescence and reflectance principles to highlight the properties of the epithelial cells. Hence, the system demonstrates an enhanced visualization of mucosal abnormalities, i.e., mouth cancer or premalignant cancerous stages that may not be visible to the human eye [29]. **VELscope^®^:** VELscope^®^ stands for Visually Enhanced Lesion scope [30]. By lesion, the vendor means any abnormality in the tissue of an organism. It works on the principle of the direct visualization of tissue fluorescence, and the variations in fluorescence intensities help the clinicians to differentiate the lesion regions from the normal area of the oral cavity. VELscope^®^ is used as an oral-cancer-screening device. Today, it is a supplementary oral examination device for non-invasive screening. With the help of VELscope, clinicians cannot determine whether the lesions are cancerous or not. Actually, it eases the detection and location of abnormal tissues that are not visible to the naked eye. When the oral cavity of a human is exposed to near ultraviolet (NUV) light, the normal cells will glow (fluoresce) brighter; on the other hand, abnormal tissues, whether cancerous or precancerous, will absorb the fluorescent light and appear darker (black). The light-absorbing property of abnormal and light-reflecting property of normal tissues of the oral cavity allow the clinicians to directly visualize the difference between the two tissues. However, a biopsy is still needed for the diagnosis of the specific detected disease.

The working principle of VELscope [31] comprises a handle-top light source attached to a holding unit for imaging. The illuminating source uses a “120 W metal halide arc (MH)” spotlight with a fundamental elongated mirror optimized for NUV (azure reflection). The flexible pinhole wheel is used to change the power attached to the light conductor. The light is attached to the light source via a holding unit with a “0.59 Numerical Aperture (NA)”. The handheld unit projects excitation rays onto the mouth cavity and a coaxial perceiving port with illumination for “fluorescence visualization (FV)”. A two-piece lens system, f = 25 mm, gathers light and transfers it to the soft muscle through a “low fluorescence excitation filter” (EX). A “dichroic mirror (DM)” is responsible for coaxial excitation and visualization paths. An “emission filter (EM)” allows the green–red glowing light to pass and stops the blue light; however, a “notch filter (NF)” splits the fluorescent light range into green and red colors.

All these devices depend on human visualization that is followed by a biopsy for the validation of the results. The effectiveness of all these devices is inconsistent [32,33]. The literature shows that with the help of these devices, currently, there is no noticeable improvement in the detection of OPMDs from the standard COE routinely performed by clinicians. These devices (VELscope^®^, Identafi) also help us to capture images of the oral cavity, but today, all the images captured via these devices are used for documentation proposes by clinicians; on the other hand, it is rather difficult for the clinicians to obtain images of the oral cavity with the Identafi. In the case of the VELscope^®^, we can mount the camera on the device; clinicians can examine the oral cavity using the camera screen and also capture the images. In order to examine these images to detect abnormalities of the oral cavity, biomedical image processing plays an important role in the diagnoses of many other cancerous diseases in different areas of the human body.

In recent years, a lot of research has been performed on the detection and identification of abnormalities inside the oral cavity using image-processing techniques [34,35,36,37]. M. Muthu Rama Krishnan et al. [38] used 114 images of oral cavities, of which 67 images were normal and the remaining were from patients suffering from oral sub-mucous fibrosis without dysplasia. All the images were captured with a “Carl Zeiss Microscope” using “H&E stained histological sections” under a “10x objective (NA 0.25)”. At a resolution of 1.33 µm and a dot size of 0.63 µm, these images were then pre-processed by using median and histogram techniques to enhance the details in the images, which was followed by the fuzzy logic technique. Later on, textural analysis was performed using wavelets and Gabor wavelets. The calculated features were than selected via Kullback–Leibler (KL). These selected features were then passed to a Bayesian classifier and Support Vector Machine (SVM) for the screening and classification of oral sub-mucous fibrosis (OSF). The results show that the SVM with the linear kernel function provides an improved classification accuracy of 92%, in comparison with the Bayesian accuracy of 76.83%.

Tze-Ta Huang et al. [39] used VELscope^®^ images of oral cancer or precancerous lesions and a control group with normal oral mucosa patients. The abnormalities in the images were chosen as the ROIs. The average intensity and heterogeneity of the ROI were calculated. A quadratic discriminant analysis (QDA) was employed to compute boundaries based on sensitivity and specificity. This differentiated the normal oral mucosae from precancerous/oral cancer lesions with a specificity of 92.3% and a sensitivity of 97.0%.

Anuradha et al. [40] proposed statistical feature extraction to classify oral cancers. They used 27 dental X-ray images to test their algorithm. The first step of pre-processing was performed using image enhancement; at the second stage, image segmentation was performed with the help of Marker Controlled Watershed segmentation, which was followed by a feature extraction method using the GLCM. These features were then passed to the SVM classifier to classify the cancer. The accuracy obtained with the help of the proposed system was 92.5%.

From the literature, it is apparent that most of the work has been performed on oral cancer diagnosis using complicated and invasive procedures such as spectroscopy and biopsies. The research gap in the classification of OPMDs and OML using machine learning techniques based on VELscope image analysis has not been addressed yet. Previous work has shown more-quantitative analysis of the intensity and heterogeneity of VELscope^®^ autofluorescence images to discriminate between cancer and precancerous cells. There is no such computer-based textural analysis system to help clinicians to find ROIs more efficiently and effectively.

### 2.2. Quadtree

**Figure 2:**
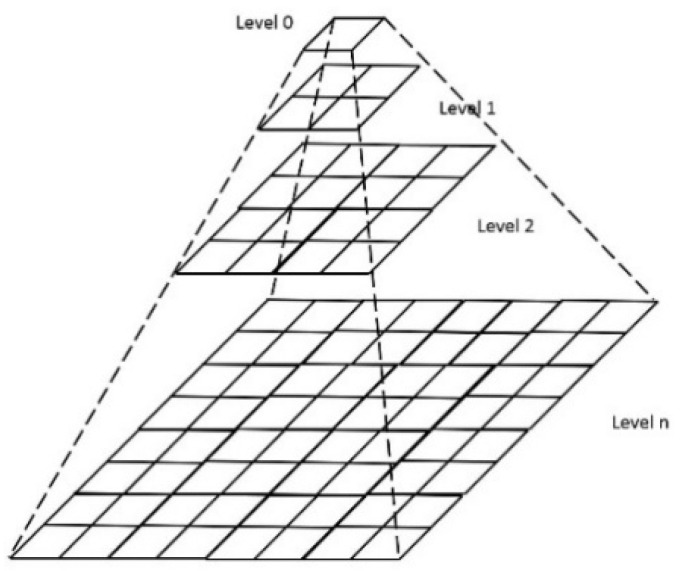
Illustration of the quadtree distribution diagram.

A quadtree is a partition of the image in which successively deeper levels represent more pleasing subdivisions of image regions, as shown in Figure 2, in which Level 0 represents the full image and Level 1 divides the above region into four equally sized regions, and this process continues until the required size of the image region is achieved. The obtained sub-image regions are called super-pixels. One of the limitations of the quadtree is that, at each level of subdivision, the size of the image must be an even number. For images that allow multiple subdivisions, a trade-off between the computation time and the resolution of the super-pixels is required. All the original images in our database have a size equal to 1792 × 1792. A quadtree division was performed to produce a 28 × 28 super-pixel matrix, where each super-pixel was of the size 64 × 64. For example, for one image, if a super-pixel size of 32 × 32 is chosen, the number of super-pixels is multiplied by 4, resulting in 6912 × 4 = 27,648 co-occurrence matrices to be computed. Hence, choosing a 64 × 64 super-pixel size ensures a reasonable computation time as well as resolution for clinicians to perform biopsies of OPMDs accurately.

### 2.3. GLCM Texture Approach

The GLCM is one of the most used textual analysis techniques that involves the statistical sampling of the gray levels that occur concerning another gray level in an image. Haralick [41,42] was the first to explain the principle of the co-occurrence probability for extracting different features in images. The GLCM works on the principle of how different combinations of pixels occur in an image or local region. In our research, fourteen parameters were used to differentiate between anomalous and standard regions.

The GLCM has drawn significant interest in recent research performed on texture analysis Hossain and Parekh [43] used a textural analysis technique for color information to recognize texture with different channel combinations, e.g., gb, rr, bb, rg, gg, rb, gr, br, and bg. Neha and Amandeep [44] used texture analysis using the GLCM to differentiate different regions (mountains, rocks, and rivers) from satellite images. Nitish and Vrushsen [45] worked on textural feature analysis for four different categories of brain tumors and classified them into different categories on the basis of different GLCM parameters. Jamesa and Dasarathy [46] performed segmentation using statistical patterns on fused biomedical images taken from different sensors, at different times, and from different viewpoints. They fused two different types of cancer and tumor images: CT and MRI images. M. Reza et al. [47] used 11,000 medical X-ray images to perform a texture analysis comparison of different types of techniques such as the local binary pattern and Canny edge operator.

## 3. Materials and Methods

### 3.1. Proposed System

**Figure 3:**
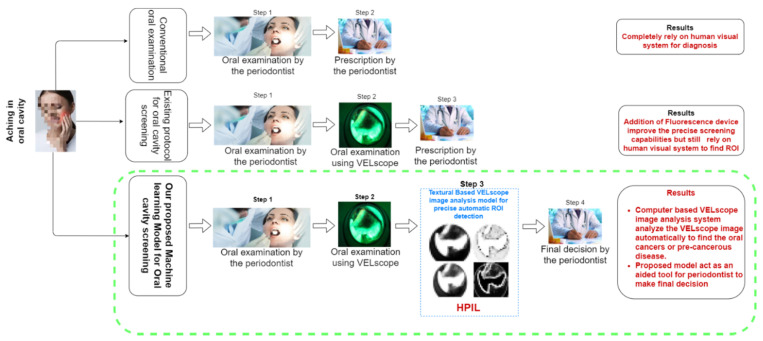
The Healthcare Professional in the loop (HPIL) workflow.

The proposed Healthcare Professional in the loop (HPIL) model acts as an aided tool for periodontists by automatically analyzing the VELscope^®^ image of an oral cavity to find the ROI more precisely. The state-of-the-textural-art model assists the periodontist with a more accurate diagnosis that results in reducing the false-positive and false-negative cases. Figure 3 depicts the overall workflow of the proposed HPIL model in comparison with the previous, existing screening procedure for the diagnosis of oral cavity abnormalities.

### 3.2. Healthcare Professional in the Loop (HPIL)

A number of authors have proposed analyzing medical images based on texture statistics or other features such as wavelets, the Gabor filter, and the fractal dimension. In this paper, the proposed method for the classification of OML and OPMDs is based on the division of images into sub-regions using a quadtree. Hence, super-pixels are created and analyzed with a GLCM textural analysis technique.

**Figure 4:**
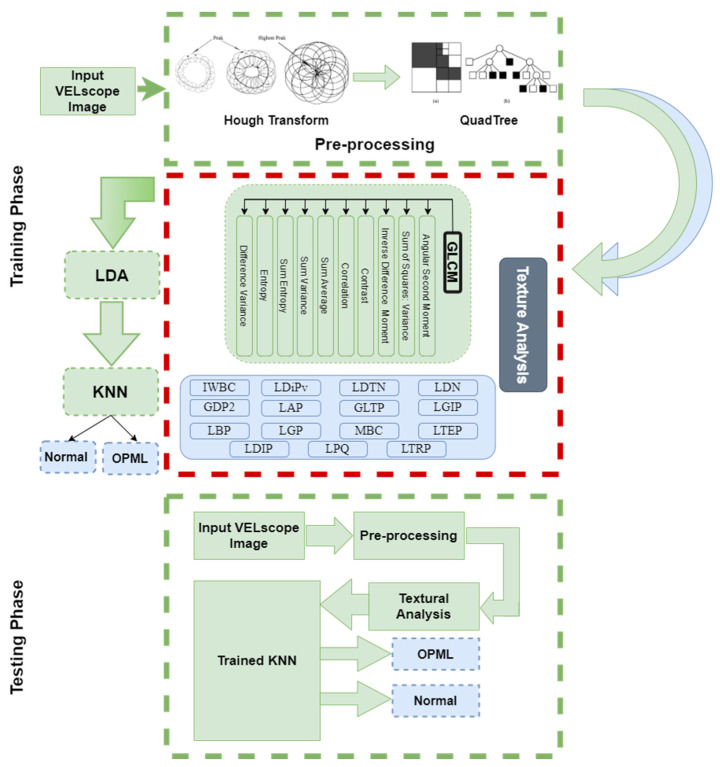
Flow chart of the textural analysis algorithm.

Figure 4 shows the overall textural analysis of VELscope^®^ images to classify the OPML and distinct regions. As the first step, an input RGB autofluorescence image is converted into a grayscale image. A pre-processing step is needed in the case of the presence of the device area in the image. The circular Hough transform (CHT) is used to detect the presence of the device part in the image. As a result, either the same grayscale image (if no device is detected) or a reduced image containing the ROI (i.e., the circular part of the image containing the inspected oral cavity area) is produced. A detailed description of CHT is provided in the “Experimental Results” section. Then, the image is subdivided into a certain number of super-pixels using the quadtree method. As the dimensions of these images are in the power of 2, it is easier to divide them into super-pixels all having the same size. Thus, the super-pixels are analyzed with the help of the GLCM to extract several features for each super-pixel. A feature selection based on linear discriminant analysis (LDA) is then performed in order to rank the GLCM features and enhance the later classification. Finally, the ranked features are classified using a k-NN classifier to identify the standard and anomalous regions (Figure 4). The performance of the algorithm was evaluated with the help of the ground truth provided by dental specialists of standard and anomalous regions of the oral cavity and expressed as the sensitivity, specificity, and accuracy.

### 3.3. Dataset of Auto-Fluorescence Images

The autofluorescence images were captured with a Cannon D620 using a VELscope^®^ device by clinicians/dental specialists. The experiments were conducted on the autofluorescence images captured by clinicians/dental specialists from the Oral Cancer Research Co-ordination Centre (OCRCC), Faculty of Dentistry, at the University of Malaya (UM). This study was approved by the Medical Ethics Committee of the Faculty of Dentistry, University of Malaya (MEC: OI DF1501/0088(L)). Twenty-two patients were involved in this experimental analysis. Out of 30, 8 subjects in our database had more than one image of the oral cavity (e.g., tongue (lift-up), lingual frenum, etc.) due to the existence of oral mucosal lesions (OML) or oral potentially malignant disorders (OPMDs) in different oral cavity regions. The images of 15 patients were captured with a 2nd generation VELscope^®^, and the remaining images were captured with a 3rd generation VELscope^®^. All the patients were suffering from a certain kind of OPMD or precancerous stage that was confirmed via histopathology. The standard and anomalous regions were appropriately defined by the clinicians, which was important and useful for performing this analysis. Twenty-two autofluorescent images were used in this study involving 24 lesions. The clinical and histopathological diagnoses of all these lesions are provided in Table 1. The ground truth for each patient image was prepared with the help of expert dental specialists (AR). The clinicians correctly delineated the standard and anomalous regions in copied images.

### 3.4. Data Acquisition Using VELscope^®^

To record the VELscope^®^ images of the oral cavity region [48,49], explicit camera settings needed to be set to obtain focused and clear images of the oral cavity.

**Figure 5:**
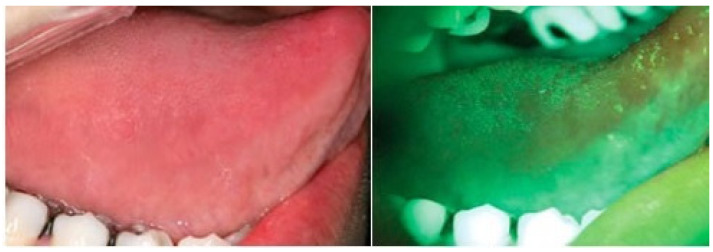
VELscope^®^ images with Canon A620 settings; the field of view (FOV) is quite straightforward and focused, as in RGB frames.

**Figure 6:**
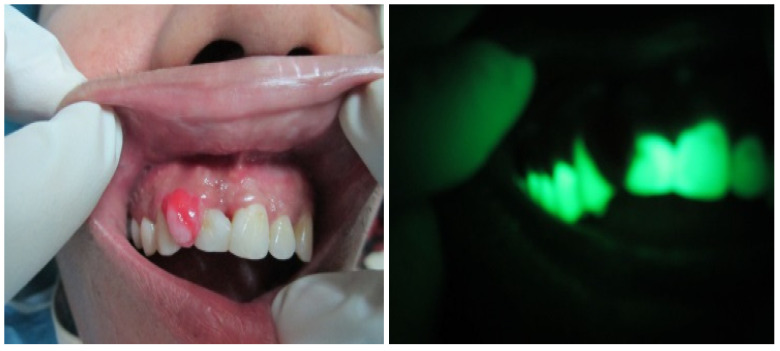
VELscope^®^ images without the Canon A620 settings; the field of view (FOV) is quite blurred and not as focused as in RGB frames.

- The first significant stage is selecting the camera as there are a number of digital cameras accessible in the shop to be adapted for use with the VELscope^®^ device, such as the Canon A620 and G7, and Nikon P5000. These single monocle reflex DSLR can be directly attached to the VELscope^®^.
- We adopted the “Canon A620” to acquire the images using the VELscope^®^. Table 2 depicts the setup configurations that research studies have used to capture VELscope^®^ images of the oral cavity. Figure 5 and Figure 6 show the effects of the oral cavity images with/without the correct settings for the “Canon A620”, respectively.

### 3.5. GLCM

In this paper, the GLCM was used to find the textural pattern of the standard and anomalous regions of the oral cavity. In this research study, we evaluated the 10 GLCM features (Angular Second Moment, Contrast, Correlation; Sum of Squares: Variance, Inverse Difference Moment, Sum Average, Sum Variance, Sum Entropy, Entropy, and Difference Variance) [41,42]; with the help of these parameters, we could perform the statistical texture analysis of the different regions of the image. These ten different statistical parameters (*f1, f2, f3….. f10*) are given below.

Whereas p(i,j) is the (i,j)th entry in the standardized gray-tone spatial-dependence matrix, $p_{x}\left(i\right)$ is the ith entry in the marginal probability matrix, $p\left(i,j\right)=\sum_{j=1}^{N_{g}}p\left(i,j\right)$. $N_{g}$ is the number of distinct gray levels in the quantized image. Similarly,$\;p_{y}\left(j\right)=\sum_{i=1}^{N_{g}}p\left(i,j\right)$, $p_{x+y}\left(k\right)=\sum_{i=1}^{N_{g}}\sum_{j=1}^{N_{g}}p\left(i,j\right)$ k = 2,3,…..$2N_{g}$, $p_{x-y}\left(k\right)=\sum_{i=1}^{N_{g}}\sum_{j=1}^{N_{g}}p\left(i,j\right)$, and k = 2,3,…..$2N_{g}-1$, [50,51,52]

**Angular Second Moment (*f*_1_)** (the Angular Second Moment is also known as the Uniformity or Energy):

$$f_{1}=\underset{i}{\sum }\underset{j}{\sum }{\left\{N\left(i,j\right)\right\}}^{2}$$

**Contrast (*f*_2_)** (processes the local variations in the gray-level co-occurrence matrix):

$$f_{2}=\overset{L_{g}-1}{\underset{n=0}{\sum }}n^{2}\left\{\sum_{i=1}^{L_{g}}\sum_{j=1}^{L_{g}}N\left(i,j\right),\left|i-j\right|=0\right\}$$

**Correlation (*f*_3_)** (measures the joint probability of occurrence of the specified pixel pairs):

$$f_{3}=\frac{\sum_{i}\sum_{j}n\left(i,j\right)-\mu_{x}\mu_{y}}{\sigma_{x}\sigma_{}}$$

where $\mu_{x}$, $\mu_{y}$,$\;\sigma_{x}$, and $\sigma_{y}$ are the means and standard deviations of $N_{x}$ and $N_{y}$.

**Variance (*f*_4_)** (sum of squares):

$$f_{4}=\underset{i}{\sum }\underset{j}{\sum }{\left(i-\mu \right)}^{2}N\left(X,Y\right)$$

**Inverse Difference Moment (*f*_5_)** (the Inverse Difference Moment is the confined homogeneity; it is high when the local gray level is uniform and vice versa):

$$f_{5}=\underset{I}{\sum }\underset{J}{\sum }\frac{1}{1+{\left(i+j\right)}^{2}N\left(i,j\right)}$$

**Sum Average (*f*_6_)**:

$$f_{6}=\sum_{i=2}^{2L_{g}}iN_{x+y\left(i\right)}$$

**Sum Variance (*f*_7_)**:

$$f_{7}=\sum_{i=2}^{2N_{g}}{\left(i-f_{g}\right)}^{2}P_{x+y}\left(i\right)$$

**Sum Entropy (*f*_8_)**:

$$f_{8}=\sum_{i=2}^{2L_{g}}N_{x+y}\left(i\right)\log\left\{N_{x+y}\left(i\right)\right\}$$

**Entropy (*f*_9_)** (provides the sum of squared elements in the GLCM, also known as the Uniformity or the Angular Second Moment):

$$f_{9}=-\sum_{i}\sum_{j}N\left(i,j\right)\log\left(N\left(i,j\right)\right)$$

**Difference Variance (*f*_10_)**:

$$f_{10}=variance\;of\;N_{x-y}$$

### 3.6. Feature Selection Based on Linear Discriminant Analysis (LDA)

The main aim of using LDA was to differentiate the set of features that provided the best discrimination between the standard and anomalous regions of the oral cavity. LDA is a mathematically robust technique and is often used to produce models that have accuracies that are as good as those of more complex methods. It is one of the powerful techniques used in classification, statistical analysis, and pattern recognition to find the best linear combination of features. It was initially developed in 1936 [53,54]. The basic working principle of LDA works on the concept of searching for linear combinations of variables (predictors) that help to separate the classes (targets). To separate the two regions, Fisher defines the scoring function as shown in Equation (1), in which $\mu_{1}$ and $\mu_{2}$ are the means of the particular regions; $\beta_{1}$ and $\beta_{2}$ are the coefficients that need to be calculated to obtain the z value.

(1) $$z=\beta_{1}\mu_{1}+\beta_{2}\mu_{2}\ldots \ldots \beta_{d}\mu_{d}$$

(2) $$s\left(\beta \right)=\frac{\beta^{T}\mu_{1}+\beta_{1}\mu_{2}}{\beta_{1}C\beta }$$

With s(β), the Score Function to be maximized, as shown in Equation (2), C is the covariance matrix, β is the coefficient matrix (linear model coefficient), and $\mu_{i}$ is the mean of the different classes; i.e., $\mu_{1}$ and $\mu_{2}$ are the represented means of two different classes, respectively.

Equation (3) can be represented in the general form of a scoring function:

(3) $$s\left(\beta \right)=\frac{z_{1}-z_{2}}{variance\;of\;z\;within\;groups}$$

Meanwhile, the linear coefficient matrix for β($\beta_{1}$ and $\beta_{2}$) can be calculated:

(4) $$\beta =C^{-1}(\mu_{1}-\mu_{2}\;)$$

The covariance matrix (*C*) can be calculated with the following mathematical Equation (5):

(5) $$C=\frac{1}{n_{1}+n_{2}}\left(n_{1}C_{1}+n_{2}C_{2}\right)$$

$n_{1},n_{2}$ represent the numbers of observations (values) in the first and second classes, respectively.

Once the coefficient and linear combination are found, the effectiveness of the discriminative vector is calculated—the Mahalanobis distance28—to calculate the difference between two regions for particular features, as shown in Equation (6).

(6) $$\Delta^{2}=\beta^{T}\left(\mu_{1}-\mu_{2}\right)$$

Δ = the Mahalanobis distance between two regions.

When the resultant value is enormous, it means there is a small overlap between the two classes. From the LDA ranking of the features, the top features are selected to perform the classification.

### 3.7. K-Nearest Neighbors (KNN) Classifier

In the early 1970s, k-nearest neighbors (KNN) was used in statistical measure analysis and the recognition of patterns [55,56,57]. It is one of the simplest and most important non-parameter algorithms that saves all the training images and classifies new test images on the basis of similarity measure analysis. One of the main advantages of using KNN is that the classification rules are produced by the case samples (classes) without the additional need for any other parameter or data.

The KNN algorithm predicts the test case category based on the training set and tries to find the most similar K number (of the nearest neighbor) of the test case. One of the simplest ways to decide for the test case is to calculate the distance of each sample and find the smallest path to predict the class of a test case.

### 3.8. Classifier Performance

**Figure 7:**
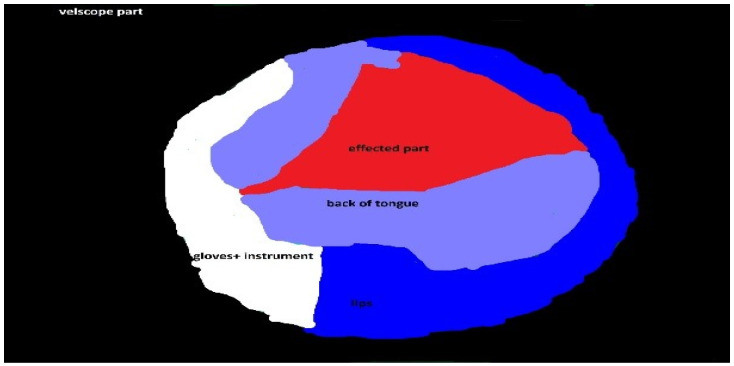
VELscope^®^ images of oral cavity annotated by the clinicians.

The efficiency of the KNN classifier for each combination of textural features was analyzed via three statistical measures, i.e., the accuracy, sensitivity, and specificity. The accuracy represents the actual results among all the numbers of regions analyzed as the system divides an image into sub-images of window 64 × 64. These sub-images are considered as anomalous regions if all their values lie in the red region, as shown in the ground truth in Figure 7. On the other hand, if a sub-image contains standard and anomalous regions, it is considered as a standard sub-image. The reason for setting this rule is to obtain an entire anomalous area for biopsy.

### 3.9. Evaluation Criteria

The evaluation was performed based on these three statistical parameters (accuracy, sensitivity, and specificity) as shown in Equations (7)–(9), respectively.

(7) $$\mathrm{Accuracy}=\frac{TP+TN}{TP+TN+FP+FN}$$

(8) $$\mathrm{Sensitivity}=\frac{TP}{TP+FN}$$

(9) $$\mathrm{Specificity}=\frac{TN}{TN+FP}$$

True Positive (TP): the anomalous disease area is properly identified by the classifier.

False Positive (FP): the standard region is identified as an anomalous region by the classifier.

True Negative (TN): the standard region is adequately identified by the classifier.

False Negative (FN): the anomalous region is identified as a standard region by the classifier.

## 4. Experimental Results

**Figure 8:**
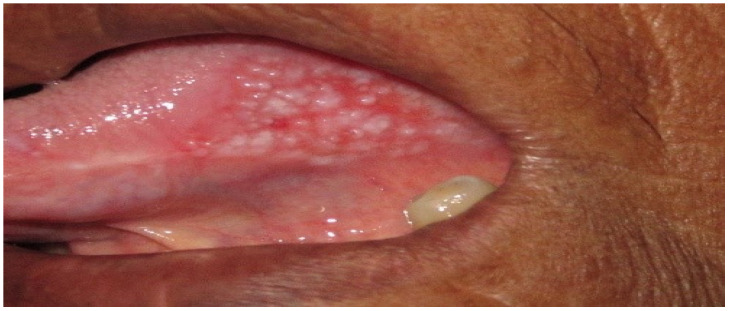
RGB image of lateral tongue.

**Figure 9:**
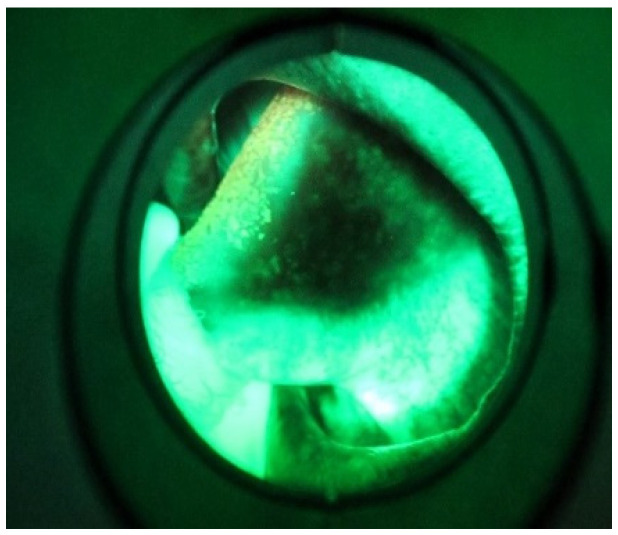
VELscope image of oral cavity.

Figure 8 shows an RGB image of the lateral tongue having mild dysplasia, but the disease area is not properly clear. Figure 9 shown a VELscope^®^ image in which the disease area appears as black, but the accurate location of the disease area is identified by the clinician, as shown in Figure 7. It is observed that the VELscope^®^ device circular region appeared in a number of images; this noisy region might affect classification accuracy. Therefore, the Hough transform was adopted as a pre-processing algorithm to remove the circular region from the VELscope^®^ images.

### 4.1. Pre-Processing (Edge Detection and CHT)

**Figure 10:**
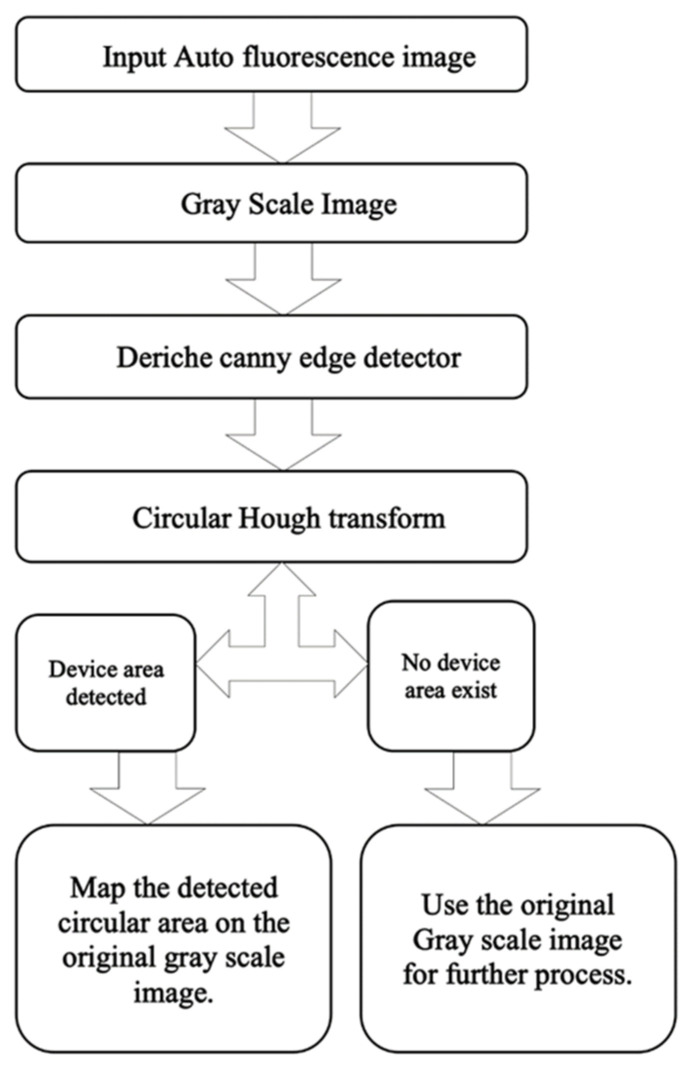
Flow chart for removing VELscope^®^ device area.

To remove the noise from the VELscope^®^ image, a pre-processing step was performed. The pre-processing was divided into two parts. First, the edges were enhanced using the Deriche–Canny detector [58], as shown in Figure 10. Later on, the CHT was applied to the edge-enhanced images. In the selected database, research has shown two cases; in the first case, the device area was present, which has been removed by CHT, and in the second case, the device area is absent.

### 4.2. Case 1: When the Device Area is Present

**Figure 11:**
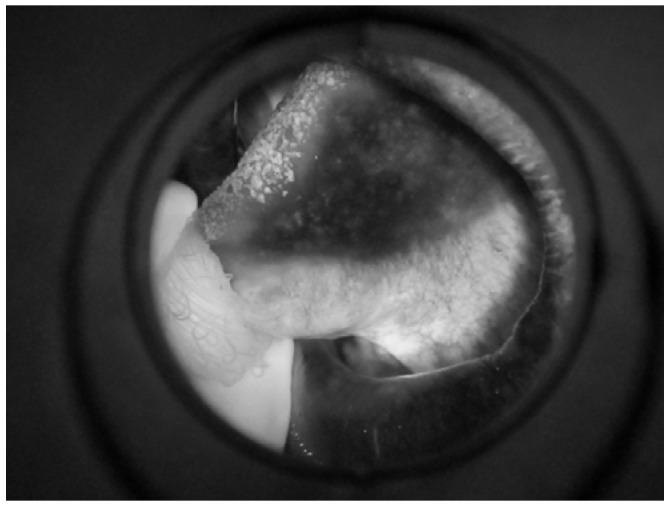
Grayscale VELscope^®^ image of an oral cavity.

**Figure 12:**
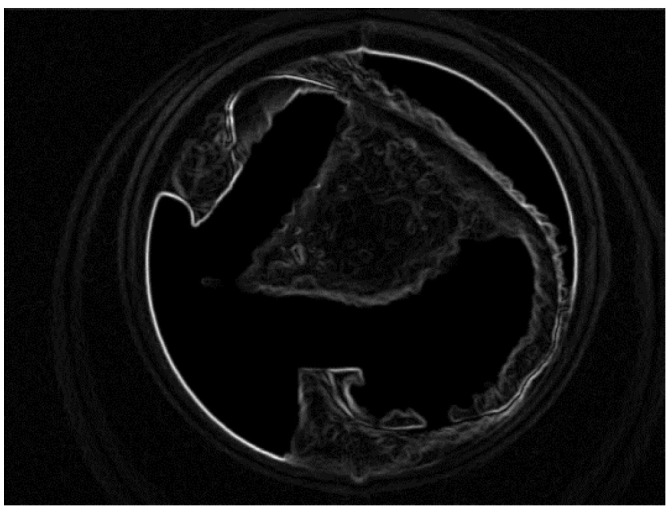
Region edge detected using Deriche–Canny edge detector.

**Figure 13:**
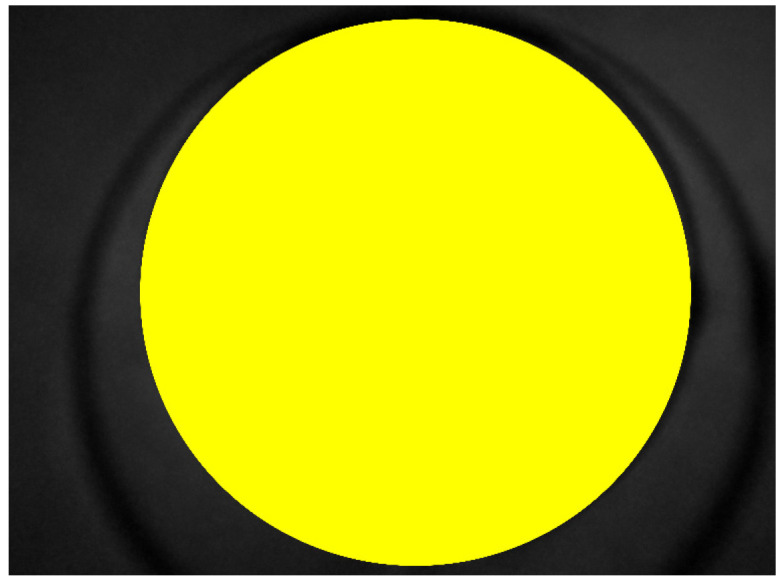
Region of interest detected using CHT.

**Figure 14:**
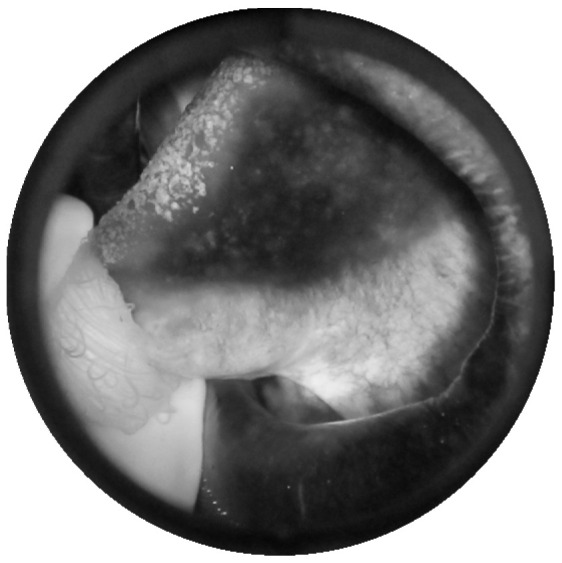
Oral cavity region extracted without the VELscope^®^ device.

Figure 11 shows a grayscale VELscope^®^ image in which the camera region appears. Initially, the grayscale VELscope^®^ images were used to detect the edges, especially the circular VELscope^®^ region, using the Deriche–Canny edge detector [59], as shown in Figure 12. The edge-detected pre-processed image was then passed to the circular Hough transform (CHT). In Figure 13, the detected circular VELscope^®^ region is shown and highlighted with a yellow-color circle; the detected region without the circular region was extracted, as shown in Figure 14.

### 4.3. Case 2: When the Device Area is Absent

**Figure 15:**
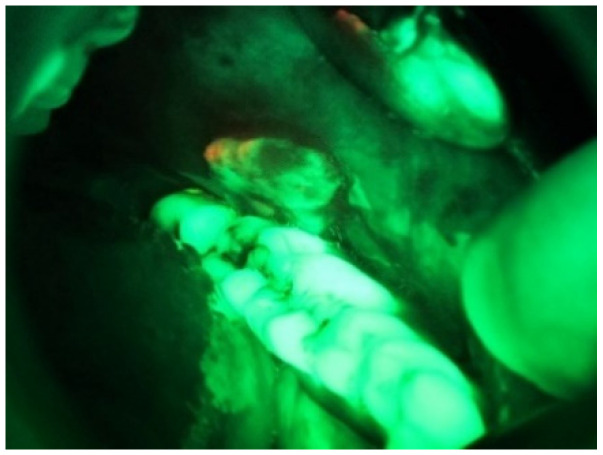
VELscope^®^ image without device area.

**Figure 16:**
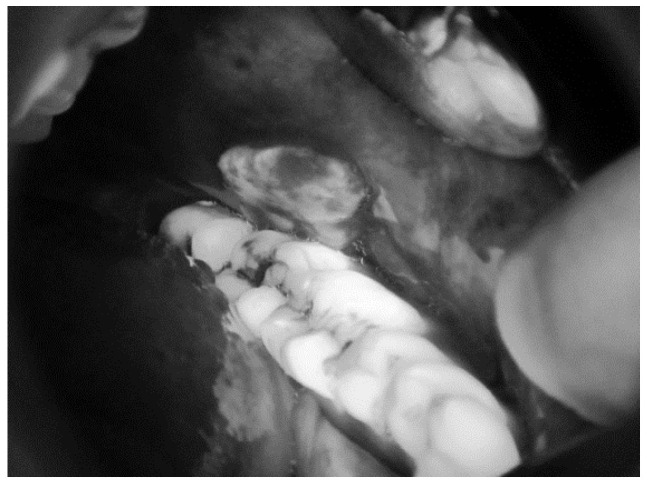
Grayscale VELscope^®^ image of the oral mucosal cavity.

**Figure 17:**
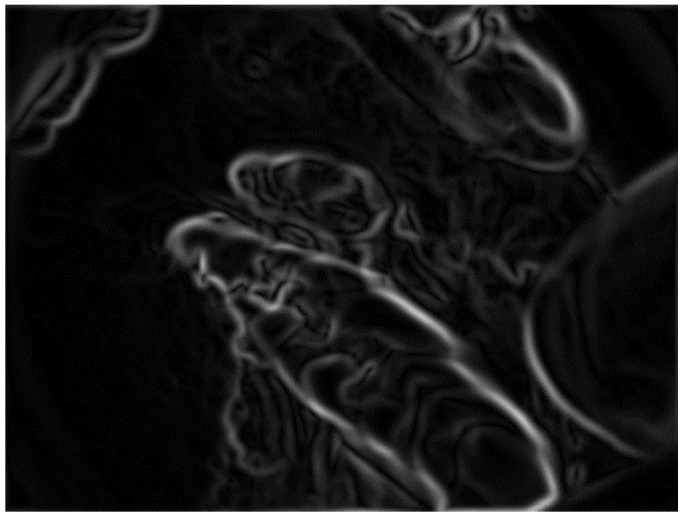
Region edge detected using Deriche–Canny edge detector.

**Figure 18:**
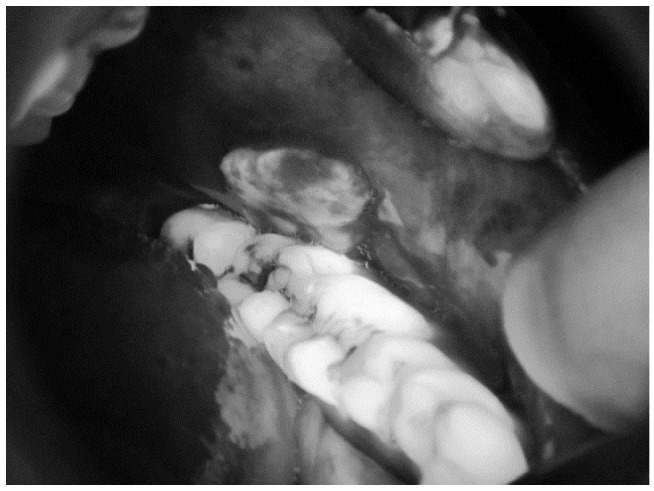
VELscope^®^ image with no circular area detected.

Figure 15 shows a VELscope^®^ image with no device area present. Initially, the RGB VELscope^®^ image was converted into grayscale, as shown in Figure 16; the grayscale VELscope^®^ images were used to detect the edges, especially the circular VELscope^®^ region, using the Deriche–Canny edge detector as shown in Figure 17. The edge-detected pre-processed image was then subjected to CHT, and CHT tried to find the circular VELscope^®^ region in an image. Figure 18 shows that no circle was detected; it depicts that the device area was absent.

**Figure 19:**
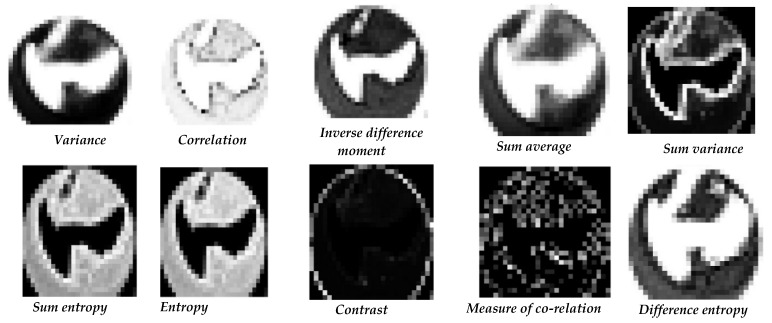
Gray-level co-occurrence matrix (GLCM) texture features of all the ten parameters using 64 × 64 quadtree size.

After this pre-processing step, the resultant image was passed to the quadtree algorithm to divide the image into small windows. Once the image was divided into super-pixels, the GLCM was applied to each super-pixel to calculate the features; a co-occurrence matrix size of 256 × 256 was calculated at 0°. This co-occurrence size for the matrix helped us in a more detailed textural analysis of each pattern in an image without rounding off values from the grayscale super-pixel. If the co-occurrence matrix size had been less than 256 × 256, then after rounding off the grayscale values to the nearest values, a lot of textural information related to the ROI might have been lost. As observed by the clinicians (project collaborators), we also know that in comparison to the standard regions, there is a fractional variation in the textural regions of OMPD. Therefore, selecting the co-occurrence size of 256 × 256 helped us to differentiate them properly, so the textural analysis technique GLCM was applied to each window of an image and 10 features were calculated, i.e., the variance, correlation, difference entropy, sum variance, sum entropy, entropy, sum average, measure of co-relation, inverse difference moment, and contrast. Figure 19 depicts the textural features obtained via the GLCM using VELscope^®^ images.

**Figure 20:**
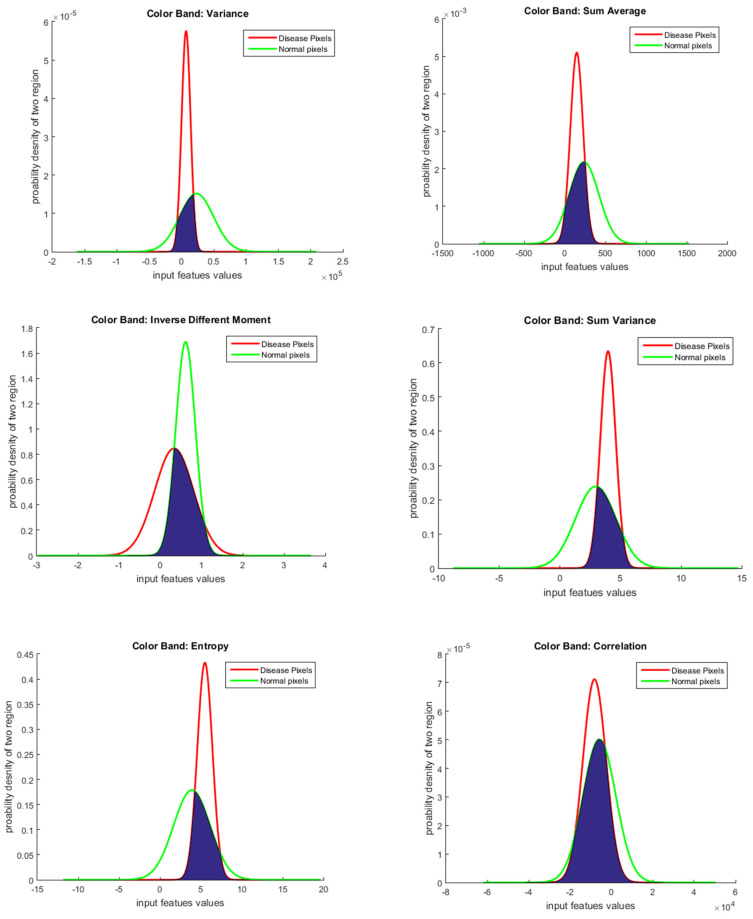

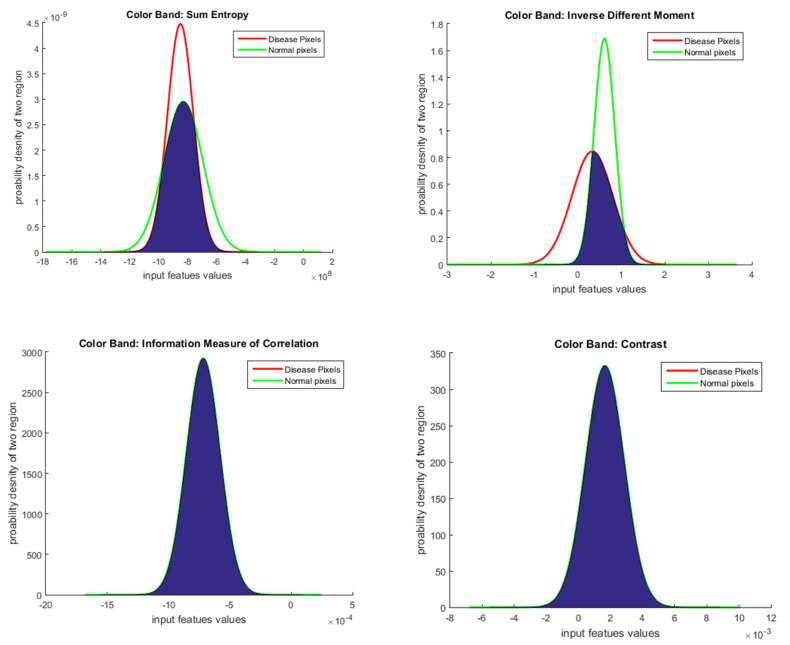
Graphical analysis of each feature selected via LDA using GLCM features.

Once all the features were calculated, the classification of the anomalous and standard regions was performed using the KNN classifier using all 10 GLCM features; however, the classification accuracy was less than 50%; in order to improve the classification accuracy, a feature selection process was performed using linear discernment analysis (LDA). Using LDA, features were arranged in descending order, as shown in Table 3, based on their capabilities of differentiating standard and anomalous regions. If the values of the features were greater, it meant that a particular feature was suitable for separate the anomalous and the standard region. In Figure 20, a graphical analysis of each feature selected via LDA using probability density phenomena is shown. These graphical results confirm that the features selected via LDA distributed the values among two regions (standard and anomalous), which would be helpful for classification. These features were then used alone as well as in combinations to find the highest accuracy with the KNN classifier, as shown in Table 3. The first eight GLCM features (the variance, correlation, inverse difference moment, sum average, sum variance, sum entropy, entropy, and difference entropy) selected using LDA provided the highest accuracy. However, the information measure of correlation and contrast features depict that the disease region and standard region overlapped with each other; these features would impact the overall system accuracy if the system used them for classification.

### 4.4. Classification

Before starting the classification using KNN, the images in the database were divided into six groups. Each group contained five images. The proposed research study performed five-fold cross validation, by training the classifier on four images, testing it on one image, and repeating this process five times for each group so that all the images passed through the testing phase.

**Figure 21:**
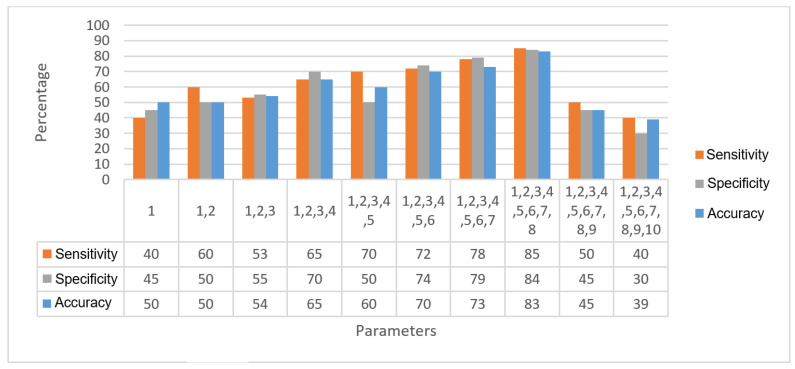
Graphical analysis of parameters vs. statistical results.

Following the ranking obtained from LDA and starting with the first parameter, at each stage, a parameter was added, and the performance was calculated using the KNN classifier. The results are shown in Table 4. The performance increased until reaching Parameter 8. From Parameter 9, the performance decreased—Figure 21 shows the graphical analysis of the parameters with the statistical results.

## 5. Discussion

Other than our proposed textural model, we evaluated the robustness and efficiency of our HPIL model in comparison with other existing texture descriptors using our VELscope^®^ database, such as gradient directional pattern (GDP) [60], gradient directional pattern 2 (GDP2), geometric local textural patterns (GLTP) [61], improved Weber local descriptor (IWLD) [62], localized angular phase (LAP) [63], local binary pattern (LBP) [64], local directional pattern (LDIP) [65], local directional pattern variance (LDiPv), inverse difference moment standardized (IDN) [66], local directional number pattern (LDNP) [67], local gradient increasing pattern (LGIP) [68], local gradient patterns (LGP) [69], local phase quantization (LPQ) [70], local ternary pattern (LTeP) [71], local tetra pattern (LTrP) [72], monogenic binary coding (MBC) [73], local frequency descriptor (LFD) [74], and local mapped pattern (LMP) [75]; however, the overall results with these textural descriptors were quite modest (as shown in Table 5) as compared to those obtained with our HPIL model, which makes it not suitable for classifying OPMD and standard regions.

Table 6 depicts the comparison of the proposed approach with the existing work. In previous research, the analysis and screening of VELscope^®^ images demanded a conventional oral examination (COE) followed by a biopsy. The existing system still relies on periodontist expertise for the annotation of the ROI to perform a biopsy. The sensitivity of the diagnosis of oral pathology ranges from 15.3% to 76%. On the contrary, the proposed textural analysis algorithm automatically analyzes the VELscope^®^ images and detects the ROI more precisely. The system achieved a more reliable sensitivity and specificity of 85 ± 5% and 84 ± 3%, respectively. Our proposed model acts as an aided tool for the periodontist to detect OPMD and precancerous cells precisely, which will result in a significant decrease in the clinician’s workload and reduce the screening time by analyzing the VELscope^®^ image automatically. Today, VELscope^®^ images are solely analyzed by the periodontist. The screening of these images demands high professional proficiency to find the exact location of OPMD or precancerous cells very precisely so that it can be used for biopsy. In addition, the labeling of VELscope^®^ images is a troublesome task for periodontists due to its tiring work routine. Currently, there is no such existing algorithm available for analyzing VELscope^®^ images to identify OPMD/OML regions automatically and help periodontists in diagnosis via biopsy.

**Figure 22:**
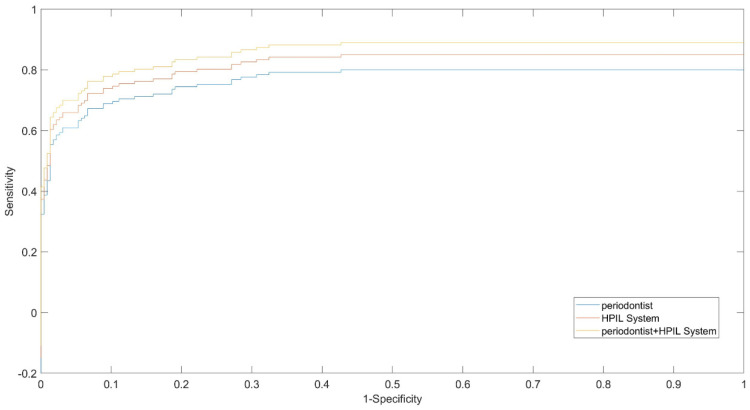
Comparative ROC between periodontist, HPIL system, and HPIL system with the periodontist.

Figure 22 shows the comparative ROC analytics for the HPIL system together with a periodontist examination, the HPIL system alone, and the consensus of two periodontists without the system. The performance of our proposed HPIL system was slightly better than the standalone periodontist examination, as displayed by the ROC graph in Figure 22, and the performance of two periodontists (sensitivity, 0.76). However, we observe that our proposed HPIL system in joint operation with a radiologist performs best out of these three scenarios. The HPIL standalone system shows slightly better performance indices (sensitivity, 0.82). On the other hand, the joint use of the HPIL system with a periodontist produced good results (sensitivity, 0.86).

**Figure 23:**
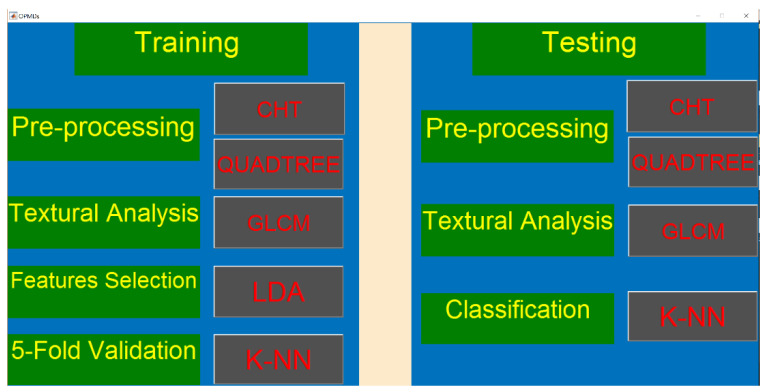
GUI for OPMDs.

A GUI was designed that will be used by the clinician for the classification of OPMDs. Figure 23 shows a graphical view of the GUI, which has been divided into a left part called “Training” and a right part called “Testing”. In the Training phase, all the dataset images are first pre-processed using the CHT and quadtree buttons. The textural patterns of these images are analyzed via the GLCM button. The results for the textural patterns of 10 different features are calculated. Once the textural patterns of all the images are calculated, the LDA button is pressed to find the best-suited features. These best-suited features are passed to the KNN classifier to validate the features selected via LDA using statistical parameters (the sensitivity, specificity, and accuracy), as shown in Table 4.

In the Testing phase, the test image is first pre-processed using CHT and quadtree. The textural pattern of this image is analyzed via the GLCM button. Once the textural patterns of all the images are calculated, the textural patterns of the features selected in training are passed to the KNN classifier to differentiate the regions, shown in the form of statistical results (sensitivity, specificity, and accuracy).

### Limitations and Future Work

This paper was limited to designing a computer-aided system for the classification of OPMDs for a biopsy. The VELscope images use digital image processing based on textural analysis representations. The motivation for using GLCM was its ability to represent the textural features in the images by calculating the co-occurrence matrix. In the future, a research study will aim at the reconstruction of the GLCM co-occurrence matrix in other directions such as 45°, 90°, and 135°; the application of the algorithm on more images of specific OPMDs representing the same disorder to classify the particular OPMD; and the analysis of other textural pattern algorithms such as LBP, Fourier, and graph cut for the classification of OPMDs, and as it is challenging to capture the images of the oral cavity using the currently existing devices such as the VELscope^®^ and Identafi^®^, these devices could be modified by fixing the camera inside the device, which would help the clinicians to capture the images along with the screening of patients. Furthermore, this study was limited to the detection of OPMDs and oral mucosal lesion regions with a relatively small dataset. The prognosis for OPMDs and precancerous and oral mucosal lesions with a bigger database including different ethnic groups will be a future aspect of our research.

## 6. Conclusions

The classification of anomalous (OML and OPMDs) and specific regions using autofluorescence images with the help of textual analysis and feature selection represents a novel way of identifying the ROI for biopsy. The method suggested in this paper involves a textural analysis of the pre-processed images to extract features, which are then ranked using LDA. Using the KNN classifier and a five-fold classifier, the best performance was obtained from the first eight features of the LDA ranking. The accuracy, sensitivity, and specificity in differentiating anomalous from standard regions of the oral cavity were 83%, 85%, and 84%, respectively. In this new area of processing oral cavity autofluorescence images, the obtained results are very encouraging. In the future, once the full dataset for OPMDs is organized, other image-processing algorithms will be used to enhance these results. Further discrimination between dysplasia and non-dysplasia in the anomalous lesions will be carried out based on GLCM texture analysis or other image-processing algorithms. These GLCM texture analyses or other image-processing algorithms for discrimination between dysplasia and non-dysplasia may further enhance the clinician’s judgment (using a COE and autofluorescence imaging) in locating the biopsy site that would best represent malignant potential.

## Figures and Tables

**Table 1:** Clinical diagnosis and histopathological diagnosis of oral potentially malignant disorders (OPMDs) in the selected database.

Diagnosis		No. of Images
**Clinical Diagnosis**	1. Homogenous leukoplakia	5
	2. Non-homogenous leukoplakia	5
	3. Oral lichen planus	9
	4. Squamous cell carcinoma	1
	5. Non-OPMDs (ulcers, abscesses, geographic tongue, frictional lesions, median rhomboid glossitis, pyogenic granuloma)	10
	Total	30
**Histopathological Diagnosis**	1. Mild dysplasia	5
	2. Moderate epithelial dysplasia	4
	3. Oral lichen planus	10
	4. Squamous cell carcinoma	1
	5. Others (ulcers, abscesses, geographic tongue, epithelial hyperkeratosis, epithelial hyperplasia, median rhomboid glossitis, pyogenic granuloma)	10
	Total	30

**Table 2:** Canon A620 camera settings to capture VELscope^®^ images.

Exposure Mode	Manual
F-Stop	5–4 (approx.)
Lens aperture	0.02
ISO	1600
Flare	zero
Focal point	manual
White balance	default

**Table 3:** GLCM feature analysis using LDA.

Parameters	Ranked LDA Measure Values	Feature Names
1	4	Variance
2	3.7	Sum average
3	3	Inverse difference moment
4	2.7	Sum variance
5	2.1	Entropy
6	1.7	Difference entropy
7	1.4	Sum entropy
8	1.1	Correlation
9	0	Contrast
10	0	Measure of co-relation

**Table 4:** Five-fold classification results using k-nearest neighbor (KNN).

Parameters #	Sensitivity	Specificity	Accuracy
1	40 ± 20	45 ± 20	50 ± 25
1, 2	60 ± 25	50 ± 20	50 ± 25
1, 2, 3	53 ± 20	55 ± 20	54 ± 15
1, 2, 3, 4	65 ± 18	70 ± 15	65 ± 17
1, 2, 3, 4, 5	70 ± 21	50 ± 24	60 ± 22
1, 2, 3, 4, 5, 6	72 ± 17	74 ± 15	70 ± 17
1, 2, 3, 4, 5, 6, 7	78 ± 10	79 ± 8	73 ± 13
1, 2, 3, 4, 5, 6, 7, 8	85 ± 5	84 ± 3	83 ± 5
1, 2, 3, 4, 5, 6, 7, 8, 9	50 ± 27	45 ± 20	45 ± 10
1, 2, 3, 4, 5, 6, 7, 8, 9, 10	40 ± 30	30 ± 15	39 ± 16

**Table 5:** Analysis of OPMD vs. typical oral cavity region using multiple textural descriptors.

Texture Descriptors	Specificity	Sensitivity	Accuracy
GDP	53 ± 20	55 ± 20	54 ± 15
GDP2	65 ± 18	70 ± 15	65 ± 17
GLTP	70 ± 21	50 ± 24	60 ± 22
IWLD	72 ± 17	74 ± 15	70 ± 17
LAP	70 ± 21	50 ± 24	60 ± 22
LBP	72 ± 17	74 ± 15	70 ± 17
LDIP	53 ± 20	55 ± 20	54 ± 15
LDIPV	65 ± 18	70 ± 15	65 ± 17
IDN	70 ± 21	50 ± 24	60 ± 22
LDNP	72 ± 17	74 ± 15	70 ± 17
LGIP	65 ± 18	70 ± 15	65 ± 17
LGP	70 ± 21	50 ± 24	60 ± 22
LPQ	72 ± 17	74 ± 15	70 ± 17
LTEP	53 ± 20	55 ± 20	54 ± 15
LTrP	65 ± 18	70 ± 15	65 ± 17
MBC	70 ± 21	50 ± 24	60 ± 22
LFD	72 ± 17	74 ± 15	70 ± 17
LMP	72 ± 17	74 ± 15	70 ± 17

**Table 6:** Comparison of our purposed algorithm with existing work.

Authors	Investigation Principle	Oral Pathology	ROI Screening ^⩔^	Statistical Analysis
Ganga et al. [76]	Conventional oral examination vs. VELscope^®^ method	Evaluate the effectiveness of the VELscope^®^ in recognizing dysplastic and/or neoplastic changes in oral mucosal lesions that were identified on conventional oral examination	Manual	Specificity = 76% Sensitivity = 76%
Scheer et al. [77]	VELscope^®^	Oral squamous cell carcinomas	Manual	Sensitivity = 33.3% Specificity = 88.6%
Farah et al. [78]	VELscope^®^	Oral potentially malignant disorders	Manual	Sensitivity = 30% Specificity = 63%
Awan et al. [32]	VELscope^®^ and conventional oral examination	Oral leukoplakia, oral erythroplakia, oral lichen planus, and oral sub-mucous fibrosis	Manual	Sensitivity = 84.1% Specificity = 15.3%
Mehrotra et al. [79]	VELscope^®^ vs. ViziLite ^®^	Oral squamous cell carcinomas	Manual	Sensitivity = 50% Specificity = 38.9%
Our proposed algorithm	VELscope^®^ vs. textural analysis approach	Oral squamous cell carcinomas, oral leukoplakia, oral erythroplakia, oral lichen planus, oral sub-mucous fibrosis, epithelial dysplasia lesions, and mild dysplasia lesions	Automatic	Sensitivity = 85 ± 5% Specificity = 84 ± 3%

^⩔^ To find region of interest (ROI): if VELscope^®^ images are analyzed by periodontist = manual; if VELscope^®^ image is analyzed by computer-aided system = automatic.

## References

[1] (2007). American Cancer Society Global Cancer Facts & Figures, Atlanta American Cancer Society. https://www.cancer.org/research/cancer-facts-statistics/all-cancer-facts-figures/cancer-facts-figures-2007.html.

[2] Williams P.M., Poh C.F.-Y., Hovan A.J., Ng S., Rosin M.P. (2008). Evaluation of a suspicious oral mucosal lesion. J. Can. Dent. Assoc..

[3] Carreras-Torras C., Gay-Escoda C. (2015). Techniques for early diagnosis of oral squamous cell carcinoma: Systematic review. Med. Oral Patol. Oral Cir. Bucal..

[4] El-Aziz A.A., Aboushousha A., Ali S., Zahran F. (2020). Prevalence of Potentially Malignant Lesions and Oral Cancer Among Smokers in an Egyptian cohort: A Hospital-based Cross-Sectional Study. Adv. Dent. J..

[5] Nagao T., Warnakulasuriya S. (2020). Screening for oral cancer: Future prospects, research and policy development for Asia. Oral Oncol..

[6] Sugerman P.B., Sabage N. (2002). Oral lichen planus: Causes, diagnosis and management. Aust. Dent. J..

[7] Velleuer E., Dietrich R., Pomjanski N., de Santana Almeida Araujo I.K., Sroka I., Biesterfeld S., Bcöking A., Schramm M. (2020). Diagnostic accuracy of brush biopsy–based cytology for the early detection of oral cancer and precursors in Fanconi anemia. Cancer Cytopathol..

[8] Kerr A.R., Robinson M.E., Meyerowitz C., Morse D.E., Aguilar M.L., Tomar S.L., Guerrero L., Caprio D., Kaste L.M., Makhija S.K. (2020). Cues Utilized by dentists in the early detection of oral cancer and oral potentially malignant lesions: Findings from the National Dental Practice-Based Reseacrh Network. Oral Surg. Oral Med. Oral Pathol. Oral Radiol..

[9] Haron N., Zain R.B., Ramanathan A., Abraham M.T., Liew C.S., Ng K.G., Cheng L.C., Husin R.B., Chong S.M.Y., Thangavalu L.A. (2020). m-Health for Early Detection of Oral Cancer in Low- and Middle-Income Countries. Telemed. E-Health.

[10] Al-Maweri S.A., Halboub E., Warnakulasuriya S. (2020). Impact of COVID-19 on the early detection of oral cancer: A special emphasis on high risk. Oral Oncol..

[11] Leuci S., Coppola N., Turkina A.Y., Bizzoca M.E., Maiorano E., Spagnuolo G., Mignogna M.D. (2020). May VelScope Be Deemed an Opportunistic Oral Cancer Screening by General Dentists? A Pilot Study. J. Clin. Med..

[12] Nikolovski B., Monevska D.P., Popovska M., Nikolovska V.R., Minovska A. (2020). Assessment of Clinical Examination Validity in Oral Cancer Risk Patients. Balk. J. Dent. Med..

[13] Tiwari L., Kujan O., Farah C.S. (2019). Optical fluorescence imaging in oral cancer and potentially malignant disorders: A systematic review. Oral Dis..

[14] Warnakulasuriya S. (2020). Oral potentially malignant disorders: A comprehensive review on clinical aspects and management. Oral Oncol..

[15] Rana M., Zapf A., Kuehle M., Gellrich N.-C., Eckardt A. (2012). Clinical evaluation of an autofluorescence diagnostic device for oral cancer detection. Eur. J. Cancer Prev..

[16] Wilder-Smith P., Ajdaharian J. (2020). Oral Diagnosis.

[17] Lingen M.W., Kalmar J.R., Karrison T.G., Speight P.M. (2008). Critical evaluation of diagnostic aids for the detection of oral cancer. Oral Oncol..

[18] Lewis B. (2019). “Oral Screening and Lesion Identification Systems”, Aetna better Heal. Pennsylvania. https://www.aetnabetterhealth.com/pennsylvania/assets/pdf/pharmacy/pharmacy-bulletins/0760%20Oral%20Screening%20and%20Lesion%20Identification%20Systems.pdf.

[19] Shrivastava N., Tyagi V. Multistage Content-Based Image Retrieval. Multistage content-based image retrieval. Proceedings of the CSI Sixth International Conference on Software Engineering (CONSEG).

[20] Perner P. (2008). Case-Based Reasoning on Images and Signals.

[21] Elizabeth D., Raj C.R., Nehemiah H., Kannan A. (2012). Computer-aided diagnosis of lung cancer based on analysis of the significant slice of chest computed tomography image. IET Image Process..

[22] Anderson S. (2004). Target classification, recognition and identification with HF radar. Symp. Target classif..

[23] Zhu X., Huang J., Zhou Q. (2011). Apparel image matting and applications in e-commerce. Proceedings of the 2011 6th IEEE Joint International Information Technology and Artificial Intelligence Conference.

[24] Zheng R., Wen S., Zhang Q., Jin H., Xie X. (2011). Compounded Face Image Retrieval Based on Vertical Web Image Retrieval. Proceedings of the 2011 Sixth Annual Chinagrid Conference.

[25] Tomita F., Tsuji S. (1990). Statistical Texture Analysis. Comput. Anal. Vis. Textures.

[26] Scott Froum D. (2015). 10 Steps to Perform an Oral Cancer Screening. Dentistryiq. https://www.dentistryiq.com/dentistry/oral-cancer/article/16350620/10-steps-to-perform-an-oral-cancer-screening.

[27] Mehrotra R., Gupta D.K. (2011). Exciting new advances in oral cancer diagnosis: Avenues to early detection. Head Neck Oncol..

[28] Niyogi S., Priyadarshini S. (2019). Non-Invasive Chairside Diagnostic Techniques: A Review. Indian J. Public Heal. Res. Dev..

[29] Bhatia N., Lalla Y., Vu A.N., Farah C.S. (2013). Advances in Optical Adjunctive Aids for Visualisation and Detection of Oral Malignant and Potentially Malignant Lesions. Int. J. Dent..

[30] Balevi B. (2007). Evidence-based decision making: Should the general dentist adopt the use of the VELscope for routine screening for oral cancer?. J. Can. Dent. Assoc..

[31] Lane P.M., Gilhuly T., Whitehead P., Zeng H., Poh C.F.-Y., Ng S., Williams P.M., Zhang L., Rosin M.P., Macaulay C.E. (2006). Simple device for the direct visualization of oral-cavity tissue fluorescence. J. Biomed. Opt..

[32] Awan K.H., Morgan P.R., Warnakulasurya S. (2007). Evaluation of an autofluorescence based imaging system (VELscope^TM^) in the detection of oral potentially malignant disorders and benign keratoses. Oral Oncol..

[33] Morgan D. (2006). Oral Surgeons and the VELscope System: Partners in Early Detection & Diagnosis. http://www.dentistrytoday.com/oral-medicine/1526.

[34] Jeng M.-J., Sharma M., Chao T.-Y., Li Y.-C., Huang S.-F., Chang L.-B., Chow L. (2020). Multiclass classification of autofluorescence images of oral cavity lesions based on quantitative analysis. PLoS ONE.

[35] Kim D.H., Kim S.W., Hwang S.H. (2020). Autofluorescence imaging to identify oral malignant orpremalignant lesions: Systematic review and meta-analysis. Head Neck.

[36] Tajiri H., Kobayashi M. (2000). 3438 Detection of early gastric cancer by a real-time autofluorescence imaging system. Gastrointest. Endosc..

[37] Feng P.-H., Chen T.-T., Lin Y.-T., Chiang S.-Y., Lo C.-M. (2018). Classification of lung cancer subtypes based on autofluorescence bronchoscopic pattern recognition: A preliminary study. Comput. Methods Programs Biomed..

[38] Krishnan M.M.R., Chakraborty C., Ray A.K. (2010). Wavelet based texture classification of oral histopathological sections. Microsc. Sci. Technol. Appl. Educ..

[39] Huang T.-T., Huang J.-S., Wang Y.-Y., Chen K.-C., Wong T.-Y., Chen Y.-C., Wu C.-W., Chan L.-P., Lin Y.-C., Kao Y.-H. (2017). Novel quantitative analysis of autofluorescence images for oral cancer screening. Oral Oncol..

[40] Anuradha K. (2013). Statistical features extraction to classify oral cancer. J. Glob. Res. Comput. Sci..

[41] Hawlick R.M. (1979). Statistical and structural approaches to texture. Proc. IEEE.

[42] Nikoo H., Talebi H., Mirzaei A. A Supervised Method for Determining Displacement of Gray Level Co-Occurrence Matrix. Proceedings of the 7th Iranian Conference on Machine Vision and Image Processing.

[43] Hossain K., Parekh R., Paruya S., Kar S., Roy S. (2010). Extending GLCM to include Color Information for Texture Recognition. AIP Conf. Proc..

[44] Sharma N., Verma A. (2013). Performance Comparison of Texture based Approach for Identification of Regions in Satellite Image. Int. J. Comput. Appl..

[45] Zulpe N., Pawar V. (2012). GLCM textural features for Brain Tumor Classification. Int. J. Comput. Sci. Issues.

[46] James A.P., Dasarathy B.V. (2014). Medical image fusion: A survey of the state of the art. Inf. Fusion.

[47] Zare M.R., Mueen A., Seng W.C. (2013). Automatic Medical X-ray Image Classification using Annotation. J. Digit. Imaging.

[48] LED Dental Inc. (2016). Fluorescence Visualization Technology. LED 0101 REV G. https://www.vineyardvalleydental.com/docs/VELSCOPE.pdf.

[49] Fourie J. (2018). VELscope: Shedding light on its ideal application. South Afr. Dent. J..

[50] Haralick R.M., Shanmugam K., Dinstein I. (1973). Textural Features for Image Classification. IEEE Trans. Syst. Man Cybern..

[51] Hussain A., Khunteta A. Semantic Segmentation of Brain Tumor from MRI Images and SVM Classification using GLCM Features. Proceedings of the Second International Conference on Inventive Research in Computing Applications (ICIRCA).

[52] Mohanaiah P., Sathyanarayana P., Gurukumar L. (2013). Image Texture Feature Extraction Using GLCM Approach. Int. J. Sci. Res. Publ..

[53] Zhang X., He D., Zheng Y., Huo H., Li S., Chai R., Liu T. (2020). Deep learning based analysis of breast cancer using advanced ensemble classifier and linear discriminant analysis. IEEE Access.

[54] Sifaou H., Kammoun A., Alouini M.-S. (2020). High-dimensional Linear Discriminant Analysis Classifier for Spiked Covariance Model. J. Mach. Learn. Res..

[55] Altman N.S. (1992). An Introduction to Kernel and Nearest-Neighbor Nonparametric Regression. Am. Stat..

[56] Temir A., Artykbayev K., Demirci M.F. (2020). Image classification by Distortion-Free Graph Embedding and KNN-Random forest. Proceedings of the 17th Conference on Computer and Robot Vision (CRV).

[57] Zhang S., Wu Y., Chang J. Survey of Image Recognition Algorithms. Proceedings of the IEEE 4th Information Technology, Networking, Electronic and Automation Control Conference (ITNEC).

[58] Bourennane E.-B., Gouton P., Paindavoine M., Truchetet F. (2002). Generalization of Canny–Deriche filter for detection of noisy exponential edge. Signal Process..

[59] Zhang Y., Li T., Li Q. (2013). Defect detection for tire laser shearography image using curvelet transform based edge detector. Opt. Laser Technol..

[60] Ahmed F., Hossain E. (2013). Automated Facial Expression Recognition Using Gradient-Based Ternary Texture Patterns. Chin. J. Eng..

[61] Orjuela Vargas S.A., Yañez Puentes J.P., Philips W. (2013). Local Binary Patterns: New Variants and New Applications.

[62] Zhang T., Jia W., He X., Yang J. (2016). Discriminative Dictionary Learning with Motion Weber Local Descriptor for Violence Detection. IEEE Trans. Circuits Syst. Video Technol..

[63] Wang X.-Y., Liang L.-L., Li Y.-W., Yang H.-Y. (2016). Image retrieval based on exponent moments descriptor and localized angular phase histogram. Multimed. Tools Appl..

[64] Yang B., Chen S. (2013). A comparative study on local binary pattern (LBP) based face recognition: LBP histogram versus LBP image. Neurocomputing.

[65] Manjunatha S.B., Guruprasad A.M., Vineesh P. (2015). Face Analysis By Local Directional Number Pattern. Int. J. Eng. Res. Gen. Sci..

[66] Clausi A.D. (2002). An analysis of co-occurrence texture statistics as a function of grey level quantization. Can. J. Remote. Sens..

[67] Rivera A.R., Castillo J.R., Chae O.O. (2012). Local Directional Number Pattern for Face Analysis: Face and Expression Recognition. IEEE Trans. Image Process..

[68] Butt M., Alkhatib W. (2015). Robust 2D Face Recognition Under Different Illuminations Using Binarized Partial Face Features: Towards Protecting ID Documents. Lect. Notes Comput. Sci..

[69] Zhou W., Yu L., Qiu W., Zhou Y., Wu M. (2017). Local gradient patterns (LGP): An effective local-statistical-feature extraction scheme for no-reference image quality assessment. Inf. Sci..

[70] Ahonen T., Rahtu E., Ojansivu V., Heikkilä J. Recognition of blurred faces using Local Phase Quantization. Proceedings of the 19th International Conference on Pattern Recognition.

[71] Satapathy S.C. (2018). Ganapati Panda Electromagnetics and Telecommunications.

[72] Murala S., Maheshwari R.P., Balasubramanian R., Maheshwari R.P. (2012). Local Tetra Patterns: A New Feature Descriptor for Content-Based Image Retrieval. IEEE Trans. Image Process..

[73] Yang M., Zhang L., Shiu S.C.K., Zhang L. (2012). Monogenic Binary Coding: An Efficient Local Feature Extraction Approach to Face Recognition. IEEE Trans. Inf. Forensics Secur..

[74] Lei Z., Ahonen T., Pietikäinen M., Li S.Z. (2011). Local frequency descriptor for low-resolution face recognition. Face Gesture 2011.

[75] Ferraz C.T., Pereira O., Gonzaga A. (2014). Feature description based on Mean Local Mapped Pattern, X Work. Visão Comput. WVC.

[76] Ganga R.S., Gundre D., Bansal S., Shirsat P.M., Prasad P., Desai R.S. (2017). Evaluation of the diagnostic efficacy and spectrum of autofluorescence of benign, dysplastic and malignant lesions of the oral cavity using VELscope. Oral Oncol..

[77] Scheer M., Fuss J., Derman M.A., Kreppel M., Neugebauer J., Rothamel D., Drebber U., Zoeller J.E. (2015). Autofluorescence imaging in recurrent oral squamous cell carcinoma. Oral Maxillofac. Surg..

[78] Farah C.S., McIntosh L., Georgiou A., McCullough M.J. (2011). Efficacy of tissue autofluorescence imaging (velscope) in the visualization of oral mucosal lesions. Head Neck.

[79] Mehrotra R., Singh M., Thomas S., Nair P., Pandya S., Nigam N.S., Shukla P. (2010). A Cross-sectional study evaluating chemiluminescence and autofluorescence in the detection of clinically innocuous precancerous and cancerous oral lesions. J. Am. Dent. Assoc..

